# The impact of social drivers, conditional cash transfers and their mechanisms on the mental health of the young; an integrated retrospective and forecasting approach using the 100 million Brazilian Cohort: A study protocol

**DOI:** 10.1371/journal.pone.0272481

**Published:** 2022-10-06

**Authors:** Daiane Borges Machado, Jacyra Azevedo Paiva de Araujo, Flávia Jôse Oliveira Alves, Luis Fernando Silva Castro-de-Araujo, Elisângela da Silva Rodrigues, Erika Fialho Morais Xavier, Rodrigo Lins Rodrigues, Davide Rasella, John Naslund, Vikram Patel, Mauricio L. Barreto

**Affiliations:** 1 Center of Data and Knowledge Integration for Health (CIDACS)- Fiocruz, Salvador, Bahia, Brazil; 2 Department of Global Health and Social Medicine, Harvard Medical School, Boston, Massachusetts, United States of America; 3 Virginia Institute for Psychiatric and Behavioral Genetics, Virginia Commonwealth University, Richmond, Virginia, United States of America; 4 Department of Psychiatry, The University of Melbourne, Melbourne, Victoria, Australia; 5 Federal University of Ceará, Ceará, Brazil; 6 Rural University of Pernambuco, Pernambuco, Brazil; PLoS ONE, UNITED STATES

## Abstract

**Background:**

Physical, emotional, and social changes, including exposure to poverty, abuse, or violence, increases youth vulnerability to mental illness. These factors interfere with development, limit opportunities, and hamper achievement of a fulfilling life as adults. Addressing these issues can lead to improved outcomes at the population level and better cost-effectiveness for health services. Cash transfer programs have been a promising way to address social drivers for poor mental health. However, it is still unclear which pathways and mechanisms explain the association between socioeconomic support and lower mental illness among youth. Therefore, we will evaluate the effect of social drivers on youth mental health-related hospitalizations and suicide, test mechanisms and pathways of a countrywide socioeconomic intervention, and examine the timing of the intervention during the life course.

**Methods:**

We will combine individual-level data from youth national hospitalization, mental health disorders and attempted suicide, suicide registries and notifications of violence, with large-scale databases, including “The 100 Million Brazilian Cohort”, over an 18-year period (2001–2018). Several approaches will be used for the retrospective quasi-experimental impact evaluations, such as Regression Discontinuity Designs, Propensity Score Matching and difference-in-differences, combined with multivariable regressions for cohort analyses. We will run multivariate regressions based on hierarchical analysis approach to evaluate the association between important social drivers (mental health care, demographic and economic aspects) on mental health-related hospitalizations and suicide among youth. Furthermore, we will perform microsimulations to generate projections regarding how mental health-related hospitalizations and suicide trends will be in the future based on the current state, and how BFP implementation scenarios will affect these trends.

**Discussion:**

The results of this project will be of vital importance to guide policies and programs to improve mental health and reduce mental health-related hospitalizations and suicide in youth. It will provide information to improve the effectiveness of these programs worldwide. If cash transfers can decrease mental health problems among youth and reduce suicide.

## Background

Mental disorders are both common in young people and persistent. Previous study estimates that the global prevalence of mental illness among children and adolescents ranges between 13–20% [1], and nearly 90% of children and youth under the age of 24 years live in low- and middle-income countries (LMICs), where they constitute up to 50% of the population [2] It is estimated that at least half, and up to 75%, of all mental health problems across the life course, emerge before the age of 24 [3]. Addressing the drivers of mental disorders during this developmental phase of the life course offers a critically important opportunity to reduce the global burden of mental disorders.

A range of social adversities and deprivations are drivers of mental illness which affect children and adolescents. Youth in low middle income countries (LMICs) are particularly vulnerable due to the higher prevalence of adversities, for example, poverty, and relatively fewer available social protections in place. There is increasing recognition of the relevance of mental health to global development strategies, in particular to the achievement of the Sustainable Development Goals (SDGs), including improving youth mental health and eradicating poverty (https://sdgs.un.org/goals). Since mental health problems affect a large proportion of youth, have profound effects on a range of social outcomes such as education and employment, and can persist into adulthood, addressing mental health problems in youth is a priority for the global health agenda and for realizing the SDGs. However, there is evidence that access to mental health services in Brazil is limited, as only 19% of children with mental illness from schools in São Paulo and Porto Alegre were reported to have had prior treatment [4].

A primary strategy for achieving the SDGs is to reduce the incidence of mental disorders, for example by targeting social determinants. Additionally, there is an ethical responsibility to address the most vulnerable young people so that existing mental health disparities can be reduced [5].

Preventing mental illness earlier during development could decrease subsequent mental health-related hospitalizations and suicide attempts. However, prevention poses a tough challenge, as these outcomes are usually the final stage of a series of small breakdowns that happen at several levels in the life course. Considering the multi-causality of the phenomenon, it is important to investigate several social drivers, risks, and protective factors at the individual and contextual level. Poverty, gender and racial inequality are associated with increased mental illness [6] and suicide [7, 8]. This might be explained by increased stress, family difficulties, alcoholism, impulsivity, violence and parental mental illnesses among the poor [3, 9–11]. However, those mechanisms are yet to be fully understood and tested, especially among youth in a LMIC setting.

Brazil has one of the largest mental health-related hospitalizations among youth, and suicide is the third most common reason for death due to external causes, and alarmingly both have increased over the last 13 years. The World Health Organisation (WHO) recommends that suicide prevention should be prioritized on the global public health agenda [12]. However, it remains unclear what interventions are best suited for targeting youth at a population level and what preventive interventions delivered earlier in life have a long-term effect on preventing mental health-related hospitalizations and suicide.

The Brazilian government introduced the Bolsa Familia Programme (BFP) in 2003 [13] to promote social inclusion and strengthen human capital among the poorest. Poverty alleviation programs could influence mental illness by diverse pathways [3, 5, 9] through decreasing income inequalities or by reducing socioeconomic hardship through increased family income, which might result in stress reduction, less family disruption, or decreased alcohol consumption [14]. Therefore, we might expect government poverty alleviation programs such as BFP to have an impact. However, whether this Brazilian government program has impacted, and knowledge of the pathways and mechanism of its impact, on youth mental illness and suicide remains unknown.

Our data of over half of the Brazilian population will help understand both (observationally) the association of multiple factors on both mental illness and suicide, and the impact of interventions targeting some of these factors. Within each broad analysis, we will explore pathways, mechanisms and interactions/moderators. This is especially important in LMICs, such as Brazil, where implementing preventative measures may be more challenging as a result of cultural and socioeconomic factors, limited resources, as also in large continental countries because of size and distribution of the population [15]. This study’s aims are as follows:

1. To use large national datasets to evaluate the effect of a broad range of social drivers and of a social-economic intervention on youth mental health,
2. To test four mechanisms and pathways (reducing parental mental illness; increasing school retention; reducing exposure to violence; decreasing impulsivity) of the social-economic intervention on reducing mental illness among youth,
3. To evaluate whether equity is a moderator of the effectiveness of the cash transfer program, e.g., whether the Brazilian cash transfer program improves the lives of the most vulnerable subgroups (with lower income, black population, and women) compared to less vulnerable groups, thereby reducing mental health disparities, and
4. Investigate early life exposures, intergenerational aspects, and to forecast the impact of socioeconomic changes and cash transfers mitigation effects in Brazil until 2030 using microsimulation models, also modelling the impact of the current COVID-19 related economic recession.

## Study innovation

The Brazilian Cash Transfer Program, “Bolsa Família Program” (BFP), is one of the largest conditional cash transfer programs worldwide, which appears to have had an important public health impact. Studies indicate that BFP has promoted social inclusion and strengthened human capital among the poorest, reducing poverty and inequalities, besides increasing school attendance among youth and reduced violence, factors that are associated with mental health [5, 7, 10, 16]. A recent study found that municipalities with higher coverage of the BFP had lower suicide, indicating that this program may have some reduction effect [7]. However, understanding the impact of this program at the individual and contextual level, analyzing by areas and municipalities, and investigating pathways and mechanisms for preventing mental health-related outcomes remains unexplored. Evaluating the impact of these changes is of great interest for health systems in LMICs such as Brazil, where dramatic socioeconomic changes have taken place in a short time. If Brazilian programs are associated with lower hospitalization from mental illness and suicide, there may be ways to support prevention through increased program coverage and integration with the current mental health strategy.

*First*, this study provides a unique opportunity to use the largest existing longitudinal dataset (The 100 Million Brazilian Cohort) in any LMIC of linked socioeconomic and health individual-level data to assess the effects of socioeconomic conditions and BFP on mental illness and suicide among young people.

*Second*, the longitudinal aspect of the cohort, with a long follow-up period of 18 years of socioeconomic and health data, will allow us to explore and assess in-depth dynamics, lagged and long-term effects of changes in living conditions, parents’ mental illness and in exposures to BFP. The long-term impact of BFP on a diverse population once such a program has been scaled up and sustained for over a decade is completely unknown.

*Third*, with this project, we will be able to identify risk factors for mental health-related hospitalizations and suicide (such as demographic factors, economic factors, parents’ underlying mental illness and substance use disorders) and investigate pathways and mechanisms using a linkage of massive datasets covering half of the country for sociodemographic information and the full country for hospitalization and mortality information.

*Fourth*, the uniquely large number of individuals of our dataset provides sufficient statistical power to assess the effects and test mechanisms of BFP on a high number of subpopulations, in terms of demographic, geographic, social, and economic conditions, thereby taking advantage of Brazil’s extreme social inequalities. To date, knowledge about the potential of Cash Transfer Programs (CCTs) on mental illness and suicide has been limited to small trials where, in general, the CCT program was not part of a national poverty alleviation strategy, and evaluations have been focused on poor populations of a few lower-income countries. This study provides the exceptional occasion to assess potential effect modification disparities, evaluating whether the Brazilian cash transfer program improved the lives of most vulnerable subgroups (with lower income, black population, and women) compared to less vulnerable groups, therefore, reducing inequalities.

*Fifth*, this study contributes to translational science with the systematic integration of the large dataset and results from retrospective impact evaluations with prospective mathematical models, developing more reliable forecast scenarios than models which use parameters from other studies or populations. The 18 year-100 million individual retrospective cohort will be used to generate projections, using microsimulation models, of an integrated synthetic cohort up to 2030, maintaining the correlation structure between variables and area/individual-specific trends in all demographic and socioeconomic variables. In creating these forecast cohorts, several alternative scenarios–including maintenance of the current poverty and inequality increase vs their gradual decrease—will be simulated, and resulting effects on mental health-related hospitalizations and suicide (as well as the policy response to it) will be evaluated.

This project provides an innovative approach to assess the effects of a large and successfully scaled program on youth mental illness and suicide. Use of a longitudinal big dataset and a pioneer integration between retrospective and forecasting evaluations, together with innovative hybrid microsimulation models, will advance our understanding of mental illness, suicide, and control measures among youth in one of the biggest countries in the world. The implications are vast, as it will help answer questions about mechanisms through which socioeconomic programs can prevent mental illness in early life. These findings will be important to other LMICs struggling to address the mental health burden and to other countries, that also faces high-income inequality, high violence rates and its continental size, which brings challenges to implementing governmental programs.

## Materials and methods

### Conceptual framework

There is a strong association between poverty and poor mental health in young people [17, 18]. Psychiatric disorders in youth are often associated with social problems such as school failure or delinquency, which increase the risk of poverty and other adverse outcomes into adulthood [3, 5]. Furthermore, mental health support is inequitably distributed, so that people living in poverty and those facing other vulnerabilities are at higher risk [5]. Therefore, youth living in LMICs have increased vulnerability to mental illness and associated high mortality rates due to their social and environmental situations [11, 19].

Economic hardship can affect mental health, increase stress, and decrease self-esteem [20]. Economic stressors have been estimated to account for 24% of suicide occurrence [21]. Income inequality is associated with increased suicide rates for both men and women, mechanisms by which this association exists include limited access to resources and increased sense of injustice for not achieving as much economic success as other people [21]. Some studies indicate that poverty is more frustrating in more unequal communities, possibly because individuals become frustrated when comparing themselves to others [22]. In Brazil, income inequality has been associated with higher suicide rates [8] Poverty can increase predisposition to mental illness such as alcohol abuse/dependence and depression in families [22]. It has been shown that cash transfer programs can improve general mental health [23] and increase hope and optimism [24]. It can also reduce factors that are associated with suicide, such as exposure to violence and impulsivity. Factors that can be tested in the current project by accessing notifications and hospitalizations related to those causes.

Several drivers and mechanisms have been hypothesized to underlie the process of the association between cash transfer and better mental health outcomes, and there is also a bidirectional and intergenerational component. Haushofer and Shapiro (2016) have shown that the receipt of a fixed monthly income resulted in a short-term increase in self-reported welfare among beneficiaries in Kenya [25]. A review summarized the causal evidence and mechanisms for the relationship between poverty and common mental illnesses [5]. Poverty can increase exposure to factors that lead to poor mental health, such as economic tension and family violence. Poverty may also be a barrier to accessing goods, resources and services (which can make it impossible to satisfy even basic needs), contributing to the sense of social injustice generated from inequities in access [22]. Therefore, cash transfers could increase the welfare of beneficiaries by providing greater financial stability, as well as improving family members mental health.

**Fig 1:**
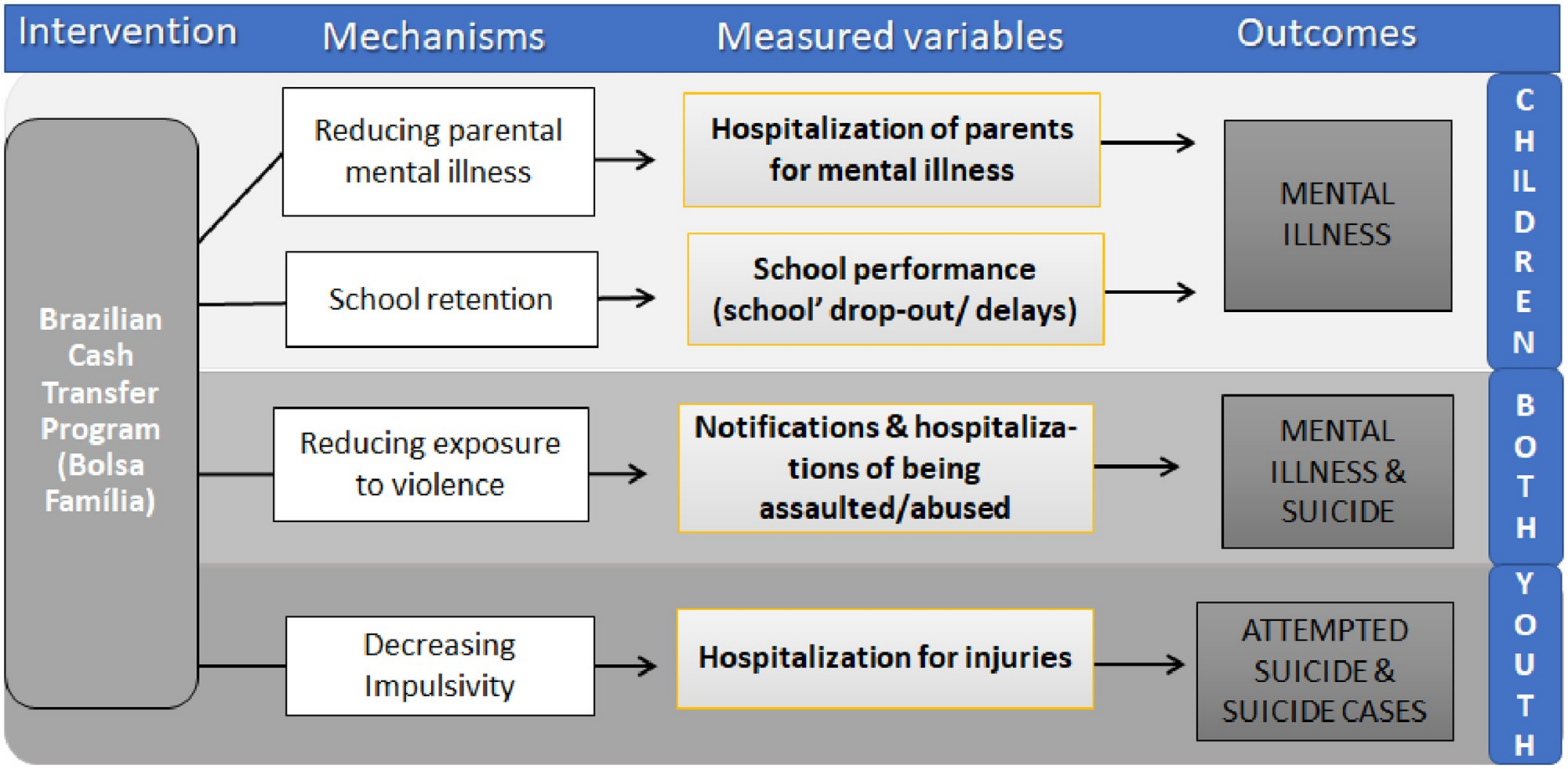
Potential mechanisms and pathways through which Bolsa Familia Program (BFP) may affect mental health-related hospitalizations and suicide among children and youth.

We hypothesize that a socioeconomic intervention, Bolsa Família Program (BFP), could be associated with better mental health outcomes among youth, by mitigating some social drivers for poor mental health, such as exposure to violence [14]. Similarly, the possible BFP effect could be mediated by the reduction of important risk factors for mental illness among youth, parents’ mental illness and school dropout because of child labor. All testable variables in our datasets. By decreasing impulsivity, measured by hospitalizations from injuries, BFP could decrease attempted suicides and suicide cases among youth (Fig 1).

Therefore, the main potential mechanisms this project will test, through which BFP may be associated with better mental health outcomes are: (i) by reducing parental mental illnesses and family separations; (ii) by improving school retention (BFP has conditionalities related to education and health); (iii) by reducing exposure to violence; and finally, (iv) by decreasing impulsivity (Fig 1).

This protocol offers a unique opportunity to test these mechanisms in a countrywide dataset, and allows subgroup analyses. Considering that the impact of those factors may happen differently among more vulnerable groups, such as black youth, women, youth with lower income and living in deprived areas, after examining the impact of cash transfers on interrupting the pathways between poverty and poor mental health through one or more mechanisms previously mentioned (e.g., improving educational outcomes, reducing impulsivity, etc). We will then evaluate the differential impact of BFP across race and gender, and how this might be explained by the mechanisms. If the effect of the cash transfer is stronger among minorities, decreasing inequalities, it can also be one of the mechanisms through which this program may improve youth mental health.

#### Data and measures

Datasets, variables, and sample sizes.

***100 Million Brazilian Cohort*.** We will analyze a cohort of subjects registered in Unified Registry for Social Programs (CadÚnico). The CadÚnico is a data collection and storage system that contains individual records with demographic and socioeconomic information on the poorer half of the Brazilian population (n = 114,007,705). Anyone who meets the eligibility criteria can be registered by visiting one of the Centers for Social Assistance (CRAS) or be registered at home, as social workers visit households periodically to identify and register eligible individuals. The criteria to be eligible for CadÚnico registration are: having a monthly family income per capita of half the minimum salary in Brazil (778 reals in 2015) or less; or having a total family income (monthly) of up to three minimum salaries. The CadÚnico contains information about housing conditions, income and demographic characteristics of all the members of a registered family [26].

***Bolsa Familia Program (BFP)*.** The Brazilian Cash Transfer Program (BFP) is the largest poverty alleviation program in the world, with about 46 million people having received the benefit up to 2015. It has three aims: income supplement guarantee for immediate relief of extreme poverty (people who live with up to 70 reals per capita) [27]; access to public services (improving education, health, and citizenship of families); and productive inclusion to increase the capacity and job opportunities, and income generation among the poorest families [28].

In 2014, the eligibility criteria for participation in the program (which is more stringent than the conditions for eligibility to register in CadÚnico) was having an income of less than 77 reals (14 USD) monthly per person or less than 154 reals in cases where there is a child, adolescent or pregnant woman in the family. The benefits were: “basic benefit”, 77 reals monthly, regardless of family composition; an extra 35 reals to families with a child or adolescent between 0–15 years, or a pregnant or nursing woman; and an extra 42 reals per adolescent from 16–17 years, in 2014. Families living in extreme poverty can accumulate all these “basic benefits” to a maximum of 1,332 reals per month (235 USD).

**Fig 2:**
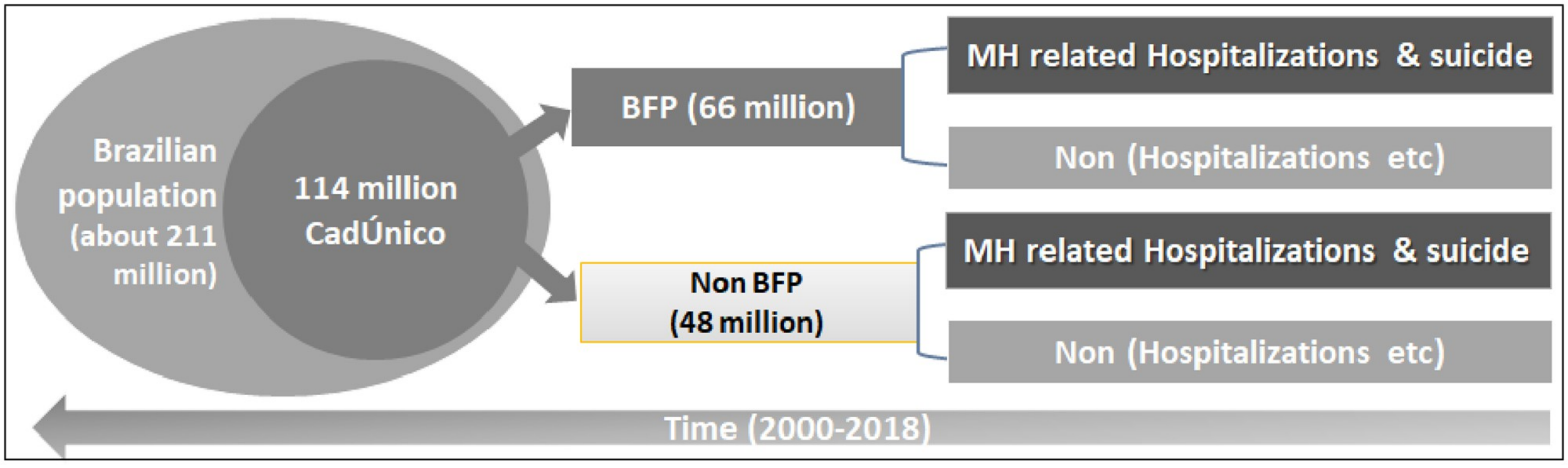
Flowchart of non-beneficiary (non-BFP) and beneficiary of the Bolsa Familia Program (BFP) to test the association between BFP and youth mental health-related hospitalizations and suicide.

All BFP beneficiaries are registered in an electronic register called CadÚnico (Unified Registry for Social Programs). Since we have both datasets available, we hold information from people who are beneficiaries in the programs and also from people who are not (see Table 1 & Fig 2) and we will be able to compare them by using robust methodologies further discussed below. A strength of the use, of a 114 million cohort, is that it covers a major part of the poor Brazilian population registered on the CadÚnico. It is especially important when we are evaluating a socioeconomic policy, and these are the people for whom the cash transfer can potentially affect the most. As we have information from all causes of death, our follow-up is more precise than other studies that cannot censor those events.

**Table 1:** Summary of the dataset’s description, from 2001 to 2018.

Dataset	Description	Sample	Variables of interest
The 100 Million Brazilian Cohort Baseline^70,73^	It was built from socioeconomic and demographic information of individuals applying for social benefits in Cadastro Unico (CadÚnico). CadÚnico is an extensive questionnaire with information on the household and on each individual.	114 million Brazilian cohort, including 66,079,181 Bolsa Familia Program beneficiaries and 47,928,524 non-beneficiaries. Among them 41,764,126, youth aged from 5–24 years old in the day of registration.	Demographic and socioeconomic variables (sex, age, ethnicity, education, work), conditions of the household (type of residence, material of household, household density, household supply, garbage destination etc), Health Care utilization, Institutional and community support (participation in groups, access to Social Assistance Reference Center, etc.) and Brazilian government social protection programs (BFP, etc).
Mortality Information System (SIM)^74,75,72^	The national system aimed to collect information on all deaths in Brazil death certificates. All deaths are recorded using a national standardized form and causes of death are registered according to the updated International Classification of Diseases CID-10.	All mortality cause (n = 21,313,290), including suicide (n = 181,527).	Demographic and socioeconomic variables (sex, age, ethnicity, education, work), place the death took place and primary and secondaries causes of death.
Hospital Information System (SIHSUS)^1^	All hospitalizations admissions financed by the Brazilian National Health System are recorded in this system. It includes about 80% of the Brazilian populations, approximately 150 million people	Hospitalizations from mental illness 5,478,980, including: 1,698,663 from alcohol and substance use-related disorders; 1,952,605 from schizophrenia; 832,974 due mood disorders, etc.	Identification and qualification of the patient, procedures, examinations, and medical acts performed, diagnosis, reasons for discharge, amounts due etc.
Notifiable Diseases Information System (SINAN)	This dataset comprises compulsory notifiable diseases information and violence, emotional and physical, as well as suicide attempt.	Compulsory notifiable diseases, including occurrences reported for: self-inflicted violence (333,933), interpersonal violence (2,111,051), and intimate partner violence (433,664).	Individual sociodemographic and clinical data of suspected and confirmed cases of interpersonal and self-inflicted violence (suicide attempt and self-agression) and intimate partner violence. It also includes information regarding the perpetrator, such as relationship with the victim.
Demographic Census (IBGE)	Household census to collect information on all households and individuals in the country and characterize the Brazilian population in terms of its demography and socioeconomic characteristics.	All Brazilian municipalities (n = 5,670).	Population, fertility, mortality, migration, housing characteristics, characteristics of the neighbourhood, education, income, labour, social inequality (Gini index), Human Development Index (HDI), proportion of poor people, etc.

Besides information from BFP, from the CadÚnico, we will also access information from the Hospitalization Information System to identify hospitalizations from: (A) mood disorders (832,974); (B) anxiety; (C) psychosis (1,952,605); (D) prescribed or illicit drug abuse and dependence; (E) alcohol abuse and dependence; (F) alcohol-related liver, problem/chronic liver disease; (G) physical injuries (228,278); (H) attempted suicide (178,606); and (I) suicide (181,527), from 2001–2018. As well as violence notifications from the Brazilian Notification System for Health Surveillance and all cause-specific mortality data, collected from the Brazilian Mortality Information System. All hospitalization, notification, and mortality information data were obtained from the DATASUS held by the Brazilian Ministry of Health and transferred to CIDACS [29] for research purposes. All the data are recorded in the three systems using the International Classification of Diseases, 10th revision. Information systems have improved considerably in Brazil; an analysis of the adequacy of mortality data from 2003 to 2005 found high quality and reliable information [30]. All the data available at CIDACS/FIOCRUZ covers a period from 2001–2018; however, we may access more recent years when the study begins.

#### Linkage of the databases

To test the pathways and mechanisms of a cash transfer program on youth mental health, we will integrate the five large individual datasets, previously mentioned, with aggregate data, analyzing the variables previously described. We have previously precisely linked the BFP data to the 100 Million Brazilian Cohort, using a social identification number (NIS). Individuals who have died by suicide, hospitalized by suicide attempt or mental disorders, or were a victim of violence (self-inflicted or interpersonal), were identified by linking Brazilian Mortality Information System (SIM), Hospitalization Information System (SIH), Notifiable Diseases Information System (SINAN) data, respectively with the 100 Million Brazilian Cohort. The 100 Million Brazilian Cohort and Health databases were linked by the Center for Data and Knowledge Integration for Health (CIDACS) [1], using Record Linkage software which was developed by CIDACS [26, 28, 31].

The linkage algorithm used five variables to identify matching records from the two databases (SIM and CadÚnico), each of which was recorded in all the datasets being linked: the beneficiary’s name, mother’s name, sex, municipality of residence code, and date of birth. The Record Linkage software performed two main steps: The record that corresponded to the data of each 100 Million Brazilian Cohort individual (the larger dataset) was indexed in the Lucene Apache library, which has a method of indexing files and performed a search of data in these files. Then for each information in the health dataset, the algorithm searched the indexed database of the 100 Million Brazilian Cohort for a potential match. A similarity calculation was performed for these records, generating a score indicating the similarities between the two linked records. The similarity calculation was performed and generated a “weighted average” which is the value of the similarity score. Finally, if the score was equal to or greater than 0.95, the link was accepted (as a correct link). In order to check the entire linked dataset, robust accuracy tests were performed, to assess the overall linkage quality. Analysis of linkage accuracy included manual verification and assessing the Receiver Operating Characteristic (ROC) curve. Details were reported elsewhere [26].

#### Analytic strategy

Aim 1: To use large national datasets to evaluate the effect of a broad range of social drivers and of a social-economic intervention on youth mental health.

In this step we will evaluate: first the effect of the BFP and social drivers such as family income, poverty, early marriage, housing characteristics, family income, parents’ divorce, family composition, and parents’ substance misuse on youth hospitalization from: (A) mood disorders; (B) anxiety; (C) psychosis; (D) prescribed or illicit drug abuse and dependence; (E) alcohol abuse and dependence; (F) alcohol-related liver, problem/chronic liver disease; (G) physical injuries; and (H) attempted suicide and (I) suicide. We will also evaluate if a family history of violence, suicide and psychiatric disorders will be associated with an increased risk of been hospitalized from a mental illness or been a suicide victim among youth. Also, if the BFP reduced the recurrence of mental health-related hospitalizations.

We will run regressions to evaluate the association between important social drivers listed above on mental health-related hospitalizations and suicide among youth in Brazil from 2001 to 2018. Multivariate regression models based on a hierarchical analysis approach will be adjusted for mental health care access, income, location of residence (urban/rural), household living conditions including household density, water supply, sewage and garbage collection, residency type and building material, and stratified by age groups, sex and race. The hierarchical models will be ordered according to the level that they act on the dependent variables (mental health hospitalizations and suicide). It is argued that mental health is conditioned by a series of proximal and intermediate factors, conditioned by socioeconomic situation, among which level of education, marital status, race, home conditions, region, and place of urban/rural residence, all considered social drivers.

To evaluate the impact of the countrywide cash transfer program on those outcomes, we will run Regression Discontinuity Design (RDD), Propensity Score Matching (PSM), and difference-in-differences, which are robust methods of impact evaluation in public health. This should help to identify the best statistical techniques to evaluate the impact of BFP on mental health-related hospitalization and suicide. Different evaluation methods make different assumptions. For instance, PSM assumes that after matching for the observed characteristics, there is no unmeasured confounding for the association between the intervention and the outcome. RDD instead assumes that people just below and just above the threshold of a continuous variable used as eligibility criteria for the intervention are “similar” in all relevant respects. Therefore, the difference in the outcome between the intervention groups is practically random near the threshold. PSM is a method that has been used in large electronic health records [10, 32]. It allows for matching where a true case-control design was not possible, which is usually the case for electronic health records of the type we will use in this project. A propensity score is a scalar summary of measured characteristics prior to treatment (potential confounders). Therefore, treated, and untreated subjects with equivalent propensity scores, also present similar pre-treatment characteristics. This method permits the generation of a grouping between intervention and no intervention that mimics the state of randomization. In our study, people who were part of one of the aforementioned programs would have specific characteristics, to control for that we will find a match for them on the remaining dataset (114 million) considering their age, sex, educational level, income, location of residence (urban/rural), household living conditions including household density, water supply, sewage and garbage collection, previous psychiatric hospitalization, residency type and building material. This allows us to inspect whether these subjects are at a higher or lesser risk of developing mental illness later in their lives. We assume that if the PSM models’ findings are similar to RDD, the results are robust given the underlying assumptions of each different model.

**Aim 2: To test four mechanisms and pathways of the social-economic intervention on reducing mental illness among youth; and Aim 3: To evaluate whether equity was a moderator of the effectiveness of the cash transfer program**, **e.g., whether the Brazilian cash transfer program improved the lives of most vulnerable subgroups (with lower income, black population, and women) compared to less vulnerable groups reducing mental health disparities.**

We will evaluate whether BFP program affects youth mental health through improving social drivers by running Regression Discontinuity Design (RDD) and Propensity Score Matching (PSM), and Structural Equation Modelling (SEM) for each of them and the outcomes. Such strategies allow us to examine the pathways by testing the impact of the intervention on variables that might be on the pathway of the relation between BFP and mental health outcomes. We hypothesize that BFP has an effect on reducing mental health-related hospitalizations and suicide among youth because it also affects their social drivers. Stratified analyses by municipality size, regions, states, and levels of Human Development Index (HDI) will also be performed.

We will include a set of covariates recognized as social drivers of mental illness for youth at the individual level such as income, income instability, unemployment, isolation and at the contextual level, variables from the municipalities where the youth live, such as population, fertility, mortality, migration, housing characteristics, characteristics of the neighbourhood, municipal income, labour, economic inequality (Gini index), Human Development Index (HDI), proportion of poor people and municipal coverage of the community mental health center and number of mental health professionals in the same municipality. We will also evaluate whether equity was a moderator of the effectiveness of the cash transfer program, e.g., whether the Brazilian cash transfer program improved the lives of most vulnerable subgroups compared to less vulnerable groups, thereby reducing mental health disparities. Therefore, we will repeat the analysis stratifying by subgroups with diverse ranges of income level, black compared with the white population, and female compared to male.

We will apply methods from the SEM group, to specify and test the paths laid out in Fig 1. SEM will be used to fit models to the specifications in Fig 1 to allow a judgement on which models best fit our data, therefore allowing the identification of the model, which might be the closest to the causal paths underlying the relationship between exposure and outcome, including potential confounders. Estimation is performed either by Maximum Likelihood. Besides identification of the best fitting models, SEM allows detailing the relationship between indicators (variables) included in the models. After deciding which models are optimal, the particular coefficient estimates are calculated, which can be of fundamental importance to the identification of the intermediate nodes that have the highest influence on the outcome, or which act as mediators or moderators. Mediators are useful representations of confounders, and as long as the path coefficients are calculated, one can precisely estimate the effects resulting from the mediator.

Aim 4: Investigate early life exposures, intergenerational aspects, and to forecast scenarios in Brazil until 2030 using microsimulation models.

Finally, to match this aim we will examine: timing of the intervention in the life course, e.g., if cash transfer delivered earlier in life (e.g., in 2004 when an adolescent was 14 years old) will be associated with lower mental health-related hospitalizations and lower suicide rates 10 years later (when the same adolescent will be 24 years old); and whether mental health-related problems such as suicidality, post-traumatic stress disorder (PTSD), violent behavior passes over to second generations; and, furthermore to forecast scenarios of mental health-related hospitalizations and suicide rates using microsimulation models. This will leverage the results from previous objectives and compare the impact of different BFP implementations on mental health-related hospitalizations and suicide in Brazil until 2030.

To answer the above sub-objectives, we propose the use of modeling for longitudinal data, since the events are observed over time. For sub-objective firstly we will apply extended Cox model [33], known as a marginal model for multiple or recurring events, to estimate whether the number of hospitalizations related to mental health increases or decreases in the course of life given whether the individuals received the intervention or not [34]. Secondly, we will run logistic regression models for longitudinal data [35] considering the age variable as a temporal measure, in which the outcome will correspond to a new binary variable in relation to having reduced or not hospitalization related to mental health (Yes/No). Regarding the intergenerational effect (sub-objective 2) on mental health issues, we will verify the influence of the parent’s mental health status on the child’s mental health through usual regression models, in which the outcome corresponds to the child’s mental health variable. Besides, we must consider a possible dependency between individuals in the same family. In this case, we will apply mixed models [36] to address the possible intra-individual correlation. All approaches will be validated using appropriate diagnostic and testing techniques.

**Fig 3:**
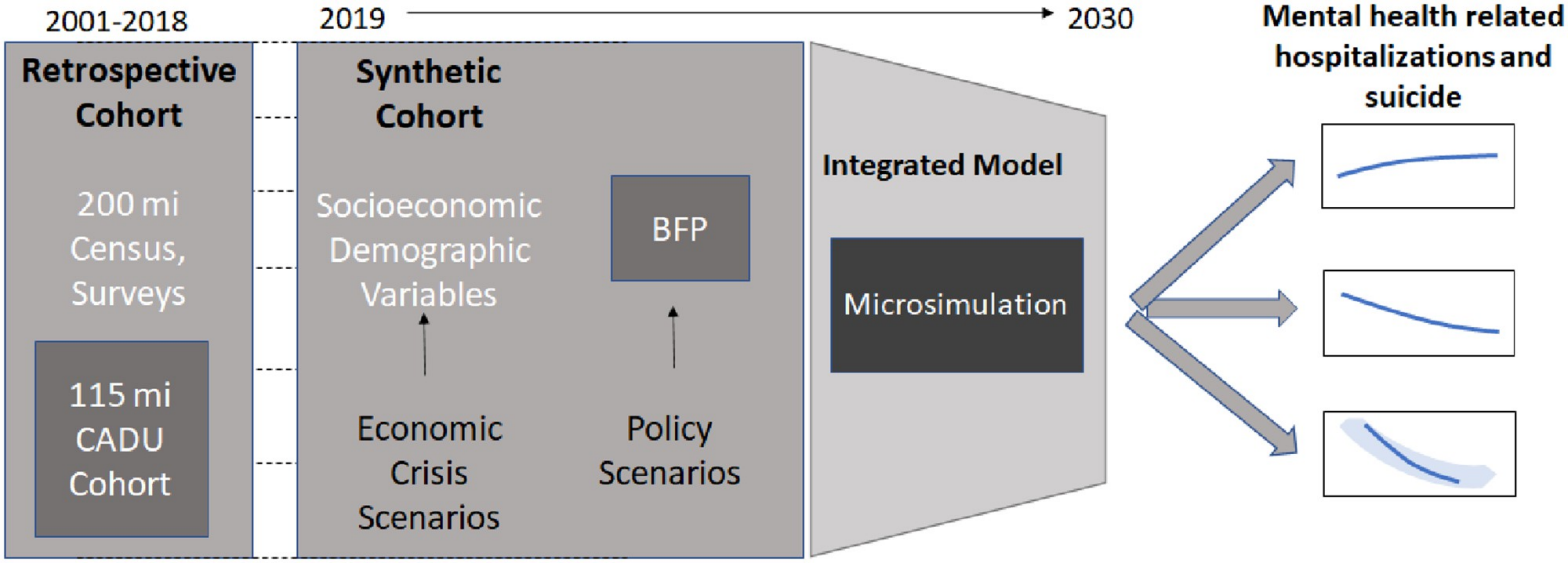
Integrated microsimulation model (MS).

The third stage of modelling will integrate the datasets and analyses above with Microsimulation Models (MS). Microsimulation is increasingly used in epidemiology and is useful to evaluate both overall and subgroup impacts of public policies [37] This is because microsimulation allows modelling of individual-specific characteristics and associated probabilities of outcomes, and when both are derived and retain the information of an existing retrospective dataset, the original correlation structure between variables and non-linear effects can be taken into account [37]. The MS integration is useful in our case, as both the heterogeneity and dynamic interactions of the population are relevant for the outcome under study. One co-investigator has developed microsimulation studies on the effect of economic crisis and BFP implementation scenarios in Brazil [38, 39] loping a platform for integration of these retrospective data and evaluations with microsimulation models for several health-related outcomes for the 100 Million Brazilian Cohort. A representation of the proposed MS model is presented in Fig 3. For the construction of this MS model, we will adopt a 2-step approach:

*Step 1*. The creation of a synthetic cohort for 2019–2030 using the demographic, socioeconomic and exposure variables as an extension of the big retrospective cohort of 2001–2018 analysis from the previous objectives. For each individual, we will simulate changes in socioeconomic variables over time based on transition probabilities and consistent with different economic scenarios. We will simulate socioeconomic changes as a result of the current economic crisis related to the COVID-19, modelling increases in poverty rates at different pace, intensity and length, together with other relevant social drivers of mental illnesses. Several BFP coverage responses will be tested through three main scenarios to reflect different economic conditions: a) a fiscal austerity scenario assuming a reduction of BFP coverage; b) a stable scenario assuming BFP coverage level is maintained at the pre-economic crisis level; and c) an enhanced coverage scenario, in which BFP is increased to protect all the newly poor individuals from the economic recession. Targeting social determinants, using cash transfer programs, could be important tools to limit hospitalizations from mental illness and suicide among youth, predicted to rise in the aftermath of economic recession consequent to the COVID-19 pandemic.

*Step 2*. The estimation of hospitalizations incidence and mortality for 2019–2030 using the individual-level values of each variable of the synthetic cohort and their effects estimates from the previous retrospective analyses. The microsimulation modelling will follow ISPOR guidelines [40] for each outcome and each scenario, 10,000 Monte Carlo simulations will be performed obtaining the Predictive Intervals of each estimate. The external validation will be performed comparing hospitalizations and mortality estimates from MS models for all country in 2001–2018 with official estimates from the Ministry of Health and from UN agencies, which are adjusted for underreporting and data quality. The linear regression of predicted versus observed values and the proportion of variance (R2) explained will be assessed, and we will verify that all observed values will be included in the 95% CIs of the simulation estimates, among other validation criteria [38–40]. Scenarios will be compared along with ratios of incidence and mortality, and the number of averted cases and deaths.

### Ethics consideration

This study will be conducted in accordance with Resolution N° 466/2012 of the National Health Council (CNS) and also complies with the international (Helsinki) research regulations. It was approved by two Ethics Committees of the: (i) Federal University of Bahia (application number: 1023107) and (ii) London School of Hygiene & Tropical Medicine (application number: 11581). The linkage of the databases will be carried out in a secure and protected environment, following a strict internal procedure to make sure data privacy and confidentiality [26]. A non-identified database will be used for the analyses, and this can only be accessed by authorized researchers, and once the data is obtained the analysis will be carried out following the CIDACS information security culture.

## Discussion

The study will use quasi-experimental approaches to assess the effect of social drivers on youth mental health-related hospitalizations and suicide, test mechanisms and pathways of a countrywide socioeconomic intervention, and examine the timing of the intervention during the life course, in a large sample of poor and impoverished Brazilian households. The BFP might result in positive impacts in all conditions related to difficulties in accessing health, education, social assistance, employment, and income, thus, improving youth mental health [4, 11, 19, 41]. The study will follow internationally recognized guidelines for conducting and disseminating the results of impact assessment studies, providing transparency in conducting data analysis, and greater comparability of results.

Suicide can be underreported due to stigma and therefore be misclassified when a family registers them. However, the process used to report violent deaths in Brazil reduces the chances of underreporting or misclassification. Suicide (and all deaths) were collected from the Brazilian Ministry of Health’s Mortality Information System (SIM) and the quality of this data has been previously recognized for having high-quality standards [30]. All death certificates in Brazil are completed following the "International Medical Certificate of Cause of Death Model", recommended by the World Health Assembly in 1948 (Brasil, 2001). Deaths due to external causes (suicide, homicide and accidents) are forwarded to the Medical Legal Institute (IML) (artigo 2º da Resolução CFM nº. 1.779/2005) where death certificates are issued and signed by an examining physician. Diagnoses are based on an autopsy, on the analyses of the circumstances in which the death occurred, personal history of the victim, and suicide risk factors [42].

Some limitations must be considered. The use of proxy in one of the four mechanisms proposed, physical injuries for impulsivity, can be a possible limitation in the analysis of mechanisms. However, assessment of impulsiveness and aggression in studies of risk factors for suicidal behavior, often depend on information from proxy informants, have been evaluated as robust [42]. Since both will be controlled by health care access, it is unlikely to have major differences between the group exposed and non-exposed. Quality of administrative data could also be challenging in these studies. Problems with quality can be related to problems with data collection and completeness of the data. However, most of the variables chosen for this project have less than 1% missing data. Most data that will be used for this proposal comes from CadÚnico that tend to be filled with precision due to the legal consequences on the beneficiaries. The forms are filled by trained interviewers, and missing information could result in not participating in the cash transfer program.

The large-scale data set will allow us to investigate comprehensively and in subpopulations the effects of BFP on youth mental health, as well test mechanisms of this effect. The use of these databases will allow exploring mental health outcomes with a high level of statistical power. The databases used in this study have national coverage, low under-registration, and some have already documented reliability. Therefore, this study will provide a comprehensive and representative analysis of the poor and extremely poor Brazilian population and reinforce the adequacy of these bases for epidemiological investigations. Results from this innovative project are expected to inform national efforts to prevent mental illnesses among youth from low-income communities in Brazil through the scale-up of economic interventions; our findings may also be relevant to other comparable middle income countries experiencing challenges, such as those resulting from the economic consequences of the COVID-19 pandemic, to address the burden of mental health problems among youth and reducing mental health disparities.

## Abbreviations

BFP: Bolsa Familia Programme
CadÚnico: Unified Registry for Social Programs
CCT: Conditional Cash Transfer
CIDACS: Center for Data and Knowledge Integration for Health
CNS: National Health Council
DATASUS: Data webpage from the Brazilian Ministry of Health
FIOCRUZ: Oswaldo Cruz Foundation
HDI: Human Development Index
LMIC: Low- and middle-income countries
MS: Microsimulation Models
NIS: Social Identification Number
PSM: Propensity Score Matching
PTSD: Post-Traumatic Stress Disorder
RDD: Regression Discontinuity Design
ROC: Receiver Operating Characteristic
SDG: Sustainable Development Goals
SEM: Structural Equation Modelling
SIH: Hospitalization Information System
SIM: Mortality Information System
SINAN: Notifiable Diseases Information System
UN: United Nations
WHO: World Health Organisation
